# DNA barcodes reveal species-specific mercury levels in tuna sushi that pose a health risk to consumers

**DOI:** 10.1098/rsbl.2010.0156

**Published:** 2010-04-21

**Authors:** Jacob H. Lowenstein, Joanna Burger, Christian W. Jeitner, George Amato, Sergios-Orestis Kolokotronis, Michael Gochfeld

**Affiliations:** 1 Department of Ecology, Evolution, and Environmental Biology, Columbia University, New York, NY 10027, USA; 2 American Museum of Natural History, Division of Vertebrate Zoology, New York, NY 10024, USA; 3 Division of Life Sciences, Department of Cell Biology and Neuroscience, and Consortium for Risk Evaluation with Stakeholder Participation *(*CRESP*)*, Nelson Biological Laboratories, Rutgers University, Piscataway, NJ, USA; 4 Environmental and Occupational Health Sciences Institute, Rutgers University, Piscataway, NJ 08854, USA; 5 Sackler Institute for Comparative Genomics, American Museum of Natural History, New York, NY 10024, USA; 6 Department of Environmental and Occupational Medicine, University of Medicine and Dentistry of New Jersey, Robert Wood Johnson Medical School, Piscataway, NJ 08854, USA

**Keywords:** mercury, *Thunnus*, sushi, DNA barcoding, seafood labelling, epidemiology

## Abstract

Excessive ingestion of mercury—a health hazard associated with consuming predatory fishes—damages neurological, sensory-motor and cardiovascular functioning. The mercury levels found in Bigeye Tuna (*Thunnus obesus*) and bluefin tuna species (*Thunnus maccoyii*, *Thunnus orientalis*, and *Thunnus thynnus*), exceed or approach levels permissible by Canada, the European Union, Japan, the US, and the World Health Organization. We used DNA barcodes to identify tuna sushi samples analysed for mercury and demonstrate that the ability to identify cryptic samples in the market place allows regulatory agencies to more accurately measure the risk faced by fish consumers and enact policies that better safeguard their health.

## 1. Introduction

Accurate identification of commercial fish species has many public health and legal applications. DNA barcodes (Hebert *et al*. 2003)—short nucleotide sequences used to identify species—can serve as an important tool allowing regulatory agencies to recognize ambiguous food items that are fraudulent or hazardous (Wong & Hanner 2008; Yancy *et al*. 2008). For tuna, DNA barcodes have been used to document market substitution, and in the case of Atlantic Bluefin Tuna (*Thunnus thynnus*), meet the requirement that species protected under the Convention on International Trade in Endangered Species of Wild Fauna and Flora (CITES) be identifiable in trade (Lowenstein *et al*. 2009; IUCN & TRAFFIC 2010). We demonstrate one of the first applications of DNA barcoding in a human health context (Cohen *et al*. 2009) by using mitochondrial DNA to identify tuna sushi to the species level concomitant with mercury testing.

Mercury methylated by microorganisms bioaccumulates, reaching high concentrations in predatory fishes such as tuna (Morel *et al*. 1998). Excessive mercury consumption is implicated in neurodevelopmental defects including mental retardation, cerebral palsy, deafness, blindness and disarthria, and adult neuro- and cardiovascular toxicity (National Resource Council 2000). Many countries have established mercury action levels above which fish may not be sold, and have also issued advisories notifying consumers of fishes high in mercury (table 1).

**Table 1.** Mercury advisory levels set by regulatory agencies. (The weekly maximum level recommended by the Food and Agriculture Organization of the United Nations and the World Health Organization for women of childbearing age is equivalent to 0.2 μg kg^−1^ body weight per day.)

agency	mercury advisory level	reference
European Commission	1.0 ppm	European Commission (2008)
Food and Agriculture Organization	1.6 μg kg^−1^ body weight per week	Codex Alimentarius Commission (1995)
Health Canada	1.0 ppm	Health Canada (2007)
Japanese Ministry of Health	0.4 ppm	Yamashita (2005)
US Environmental Protection Agency	0.1 μg kg^−1^ body weight per day	EPA (1997)
US Food and Drug Administration	1.0 ppm	FDA (2000)
World Health Organization	1.6 μg kg^−1^ body weight per week	Codex Alimentarius Commission (1995)

Owing to relaxed international labelling requirements set by the United Nations Food and Agriculture Organization (FAO), species descriptions are often inaccurate, disputed by nations or missing (Jacquet & Pauly 2008). Many countries have ambiguous or no species-specific labelling requirements such as the US where the approved market name for all members of *Thunnus* in addition to Frigate Tuna (*Auxis thazard*), Kawakawa (*Euthynnus affinis*), Skipjack Tuna (*Katsuwonus pelamis*) and Slender Tuna (*Allothunnus fallai*) is ‘tuna’ (FDA 2008). Tuna sushi, or *maguro* in Japanese, is made from five species sometimes specified in restaurants as bluefin tuna (*T. maccoyii*, *T. orientalis*, or *T. thynnus*), Bigeye Tuna/ahi (*T. obesus*) or Yellowfin Tuna/ahi (*T. albacares*; Lowenstein *et al*. 2009). Because of overlap in appearance and taste (Catarci 2005), molecular identification is one of the most precise methods for identifying tuna in the marketplace (Lowenstein *et al*. 2009; Viñas & Tudela 2009). In the context of mercury analysis, DNA barcodes enabled us to determine which species warrant inclusion in consumer advisories or trade restrictions, and whether the data used by health agencies reflect accurately the mercury threat faced by consumers.

## 2. Material and methods

We tested the mercury content of 100 tuna sushi samples from 54 restaurants and 15 supermarkets collected from October 2007 to December 2009 in New York, New Jersey, and Colorado. The New York Times collected 20 samples, and we collected the rest. We identified them using nucleotide characters and BLASTN against NCBI GenBank (see the electronic supplementary material, table S1) using the cytochrome *c* oxidase subunit I (*cox1*) gene sequence, following the methodology detailed in Lowenstein *et al*. (2009). Because the three closely related species of bluefin are often not differentiated in global trade (Catarci 2005) we pooled these data into one category for the mercury analysis. We further categorized samples according to whether they were sold as lean red tuna (*akami* in Japanese) or fatty tuna (*toro*) because mercury and lipid concentrations are inversely proportional in tuna (Balshaw *et al*. 2008*a*).

To measure total mercury, a 2 g (wet weight) subsample of fish tissue was digested in trace metal grade nitric acid (Fisher Chemical) in a microwave (CEM, MDX 2000), using a digestion protocol of three stages of 10 min each under 50, 100 and 150 pounds per square inch (3.5, 7 and 10.6 kg cm^−2^) at 80 per cent power. Digested samples were subsequently diluted to 25 ml with deionized water. All laboratory equipment and containers were washed in 10 per cent HNO_3_ solution and rinsed with deionized water prior to each use. Mercury was analysed by cold vapour technique using a Perkin Elmer FIMS-100 mercury analyser, with an instrument detection level of 0.004 μg g^−1^ and a method detection level of 0.010 μg g^−1^. All samples were tested twice, and 15 samples with the highest mercury levels were tested three times. For 98 of the samples results from all runs were within 5 per cent, and two samples within 10 per cent. As a control, we used the National Institute of Standards and Technology dogfish muscle trace metal reference material (DORM-2) alongside the samples and our results were always within the total mercury certificate range (4.38–4.90 ppm). For sushi-grade tuna, approximately 97 per cent of total mercury is methylmercury (Hight & Cheng 2006). The mercury analysis was carried out at Rutgers University and the genetic identification at the American Museum of Natural History with no prior knowledge of sample identity or mercury concentration.

Statistical analyses were performed on GraphPad Prism v. 5.00b for Mac OSX (www.graphpad.com). Before using parametric tests, we performed a log(*x* + 1) transformation on all mercury data used in this study and assessed conformation to a normal distribution using the D'Agostino–Pearson omnibus test (*α* = 0.05). We assessed homogeneity of variances for the one-way ANOVA using Bartlett's test, as well as for comparisons of two groups using an *F*-test (*α* = 0.05).

## 3. Results

Mercury concentrations varied significantly across sample categories (one-way ANOVA: *F*_4,95_ = 11.81, *p* < 0.0001; table 2). The mercury levels in bluefin *akami* and all Bigeye Tuna samples were significantly higher compared with bluefin *toro* and Yellowfin Tuna *akami*. The mean mercury concentrations of all samples exceed the concentration permitted by Japan (Yamashita *et al*. 2005), and the maximum daily consumption considered safe by the US Environmental Protection Agency (EPA 1997). Mean mercury levels for bluefin *akami* exceed those permitted by the US Food and Drug Administration (2000), Health Canada (2007) and the European Commission (2008). On average, one order of Bigeye Tuna sushi—the species used most often for sushi (Catarci 2005)—exceeds the safe maximum daily dose recommended by Health Canada (2007) and the safe limit established by the World Health Organization and FAO for women of childbearing age (Codex Alimentarius Commision 1995).

**Table 2.** Total mercury (Hg) content in tuna sushi samples. (Data from samples identified as one of the three species of bluefin (*T. maccoyii*, *n* = 7; *T. orientalis*, *n* = 4; *T. thynnus*, *n* = 18), were pooled into a single category. *Akami* is the Japanese word for lean red tuna, and *toro* for fatty tuna. Total mercury (ppm) varied significantly across sample categories (one-way ANOVA: *F*_4,95_ = 11.81, *p* < 0.0001). Categories assigned as ‘a’ were significantly different from those assigned ‘b’ (Tukey's multiple comparison test). The mean dose was calculated for the default weight of a 60 kg adult woman (WHO 1972; Health Canada 2007) consuming a single order.)

	total Hg (ppm)								
sample category	mean	median	s.d.	min	max	assignment	mean dose of total Hg (μg kg^−1^ body weight per day)	sample mass (g; mean ± s.e.)	sample size
Bigeye Tuna akami	0.871	0.794	0.393	0.336	1.716	a	0.344	22.48 ± 2.843	36
Bigeye Tuna toro	0.989	0.685	0.716	0.365	2.254	a	0.351	20.82 ± 2.941	9
bluefin tuna akami	1.043	1.028	0.478	0.368	1.916	a	0.180	12.09 ± 2.046	10
bluefin tuna toro	0.385	0.307	0.244	0.166	1.027	b	0.123	21.18 ± 2.428	19
Yellowfin Tuna akami	0.474	0.435	0.294	0.095	1.377	b	0.164	18.34 ± 2.823	26

**Figure 1.**
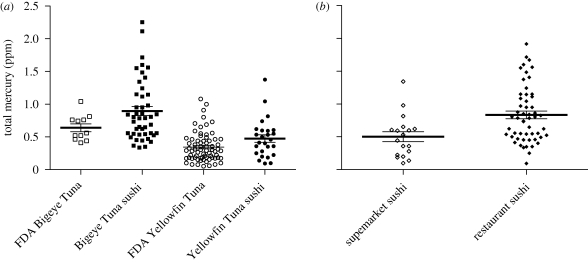
(*a*) Total mercury concentration (ppm; mean ± s.e.) in Bigeye Tuna samples (squares) and Yellowfin Tuna (circles) collected by the US Food and Drug Administration (FDA; unfilled) and sushi samples collected for this study (filled). The FDA lacks data on mercury levels in bluefin tuna. Sushi samples represent both *akami* (lean red tuna in Japanese) and *toro* (fatty tuna in Japanese). (*b*) Mean total mercury in *akami* sushi samples sold in supermarkets (unfilled diamonds) and restaurants (filled diamonds) for all species. No *toro* was found in supermarkets.

As documented previously for Southern Bluefin Tuna (Balshaw *et al*. 2008*a*), we found significantly less mercury in bluefin *toro* than in *akami* (*t* = 5.109, *p* < 0.0001), but no significant difference for Bigeye Tuna (*t* = 0.363, *p* = 0.717). We found no significant difference in bluefin mercury levels comparing data from a study (Storelli *et al*. 2002) with greater sampling (*n* = 161, mean = 1.18 ppm, s.d. = 0.85) to our bluefin *akami* results (Mann–Whitney *U*-test, *p* = 0.59). The total mercury levels we found in Yellowfin Tuna sushi was significantly higher (Mann–Whitney *U*-test, *p* = 0.0236) than in samples obtained by the FDA (2004), as was the case for Bigeye Tuna (*t*-test with Welch's correction, *t* = 2.549, *p* = 0.0162; figure 1). Finally, we found that the concentration of total mercury was also higher in our samples sold in restaurants compared with supermarkets (*t* = 3.249, *p* = 0.0018; figure 1).

## 4. Discussion and conclusions

Our results demonstrate the use of DNA barcodes to enable regulatory agencies to identify unknown and potentially hazardous samples. A multi-locus genetic species identification method was recently proposed for tuna (Viñas & Tudela 2009), and while we agree that multi-locus approaches perform better in cases of introgressive hybridization, this discussion does not have a negative impact on our findings presented here.

Mercury concentrations in tuna are positively correlated with body size (Storelli *et al*. 2002; Yamashita *et al*. 2005), and larger individuals are more likely to be sushi-grade and valued the highest (Catarci 2005). The finding that the mercury levels in Bigeye Tuna *akami* and *toro* were not significantly different may be owing to the fact that premium Bigeye *toro* cuts on average have half the fat content of bluefin (Shimamoto *et al*. 2003; Balshaw *et al*. 2008*a*) and because larger fish typically have more belly fat and are preferentially selected for *toro*. Furthermore, whereas thousands of tons of bluefin per year are fattened in farms prior to export (Catarci 2005), which can also reduce mercury (Balshaw *et al*. 2008*b*), the vast majority of Bigeye Tuna are harvested directly from the wild. Because the mercury concentrations found in our sushi were significantly higher than levels documented by the Food and Drug Administration (FDA) (figure 1), this could reflect that our samples came from larger fish (the FDA lacks bluefin data). We found significantly lower mercury levels in supermarket sushi (figure 1) because samples were dominated (77%) by Yellowfin Tuna, which comprised a minority of restaurant samples (22%; χ^2^ = 18.14, *p* < 10^−4^) and was found to be the species with the lowest mercury concentration (table 2). By allowing for the direct measurement of samples collected from the marketplace, DNA barcoding has the potential of revealing mercury measurements more reflective of the threat faced by consumers allowing for the enactment of policy that better safeguards consumer health.

Our results suggest health agencies should consider adding Bigeye and bluefin tuna to mercury advisories. For instance, the mercury levels in these species are within the bounds of fish the FDA and EPA advise pregnant or nursing women and children to avoid entirely (EPA & FDA 2004), and thus these tunas should be included in the advisory. Consumers could make more informed health decisions if the FDA, and regulatory agencies in other nations, enforced market-specific names for species high in mercury.
